# Endoperiodontal lesions: diagnosis first, then treatment and not always tooth extraction: a cross-sectional survey in Spain and a proposal of a clinical treatment protocol

**DOI:** 10.4317/jced.61130

**Published:** 2024-01-01

**Authors:** Paula García-Tuñón-Pérez, Aitziber Fernández-Jiménez, Irene Lafuente-Ibañez-de-Mendoza, Ruth Estefanía-Fresco, Xabier Marichalar-Mendia, Ana-María García-De-La-Fuente

**Affiliations:** 1 Degree in Odontology. University of the Basque Country (UPV/EHU), Biscay, Spain; 2 Ph.D., Associated professor, Research Group: GIU21/042. Department of Stomatology, University of the Basque Country (UPV/EHU), Biscay, Spain; 3 Ph.D., Associated teacher, Research Group: GIU21/042. Department of Stomatology, University of the Basque Country (UPV/EHU), Biscay, Spain; 4 Ph.D., Professor, Research Group: GIU21/042. Department of Nursery I, University of the Basque Country (UPV/EHU), Biscay, Spain; 5 Ph.D., Professor, Research Group: GIU21/042. Department of Stomatology, University of the Basque Country (UPV/EHU), Biscay, Spain

## Abstract

**Background:**

Endoperiodontal lesion (EPL) is defined as a pathological communication between pulpal and periodontal tissues. Currently, accurate diagnosis and treatment of this pathology are challenging. This study aims to identify the different endoperiodontal therapies to propose a clinical protocol to simplify and unify the criteria for EPL treatment.

**Material and Methods:**

Observational cross-sectional study through an electronic survey. This study matches STROBE guidelines. The anonymous questionnaire contained open-ended and close-ended questions and was distributed to dentistry professors of the UPV/EHU and different professionals from Spanish associations and scientific societies. The data collected were analyzed using descriptive and analytical statistics.

**Results:**

A total of 128 responses were obtained, of which 120 were active professionals or had not been so for less than 5 years. The majority of professionals were women (65.6%) and from the Basque Country (63.9%). A total of 86.6% reported having complementary studies to a degree or a bachelor’s degree. The treatments performed by these professionals were similar to those reported in the literature, which started with root canal treatment when there was an endodontic origin (91.5%), and with basic periodontal treatment when periodontal (51.3%).

**Conclusions:**

Considering the current scientific evidence and the clinical practice of professionals in the treatment of EPL, we designed a clinical protocol. This protocol needs validation in larger populations and with longer follow-ups.

** Key words:**Clinical protocol, Dental pulp diseases, Periodontal diseases, Review, Surveys and questionnaires.

## Introduction

Endoperiodontal lesion (EPL) is currently defined as the acute or chronic pathological communication between pulpal and periodontal tissues, which may have originated in the apical periodontium, in the lateral periodontium or as a combined lesion between the two biological spaces (1). This communication can occur via anatomical or non-physiological pathways (2,3).

Multiple classifications of EPL have been proposed over the years, the origin-based one by Simon *et al*. (4) being one of the most widely used. However, the common symptomatology of the lesions, including inflammation or pulp necrosis, increased probing depth (PD) (5), pain, and extensive radiographic bone loss that may reach the apex (6), hinder the diagnosis of the primary origin. In 2018, the American Association of Periodontology (AAP) and the European Federation of Periodontology (EFP) classified EPL according to the presence or absence of root damage (1).

Regarding treatment sequence, pulp vitality and periodontal involvement must be assessed; thus, when facing a negative dental pulp test, the first step is to perform a root canal treatment (7), followed by an adequate periodontal phase (8). Sadly, there is scarce evidence regarding the diagnosis and treatment of these lesions and there is no established and standardized clinical protocol for clinicians to treat these patients. Furthermore, to our knowledge, there are no studies assessing the clinical experience of dental professionals on the diagnosis and treatment of EPL.

With this background, hereby we identify what sequence of treatment the professionals follow in their daily clinical practice when treating an EPL, by means of a survey specifically designed for this study, in order to propose a standardized clinical protocol, based on our results and the scientific evidence.

## Material and Methods

This is an observational cross-sectional study using an electronic survey designed by the authors (“Google Forms” platform), which was approved by the Ethics Committee of the University of the Basque Country (UPV/EHU) (143/2021) and matches STROBE guidelines (Strengthening the Reporting of Observational Studies in Epidemiology) (9).

-Study design

A questionnaire with one open-ended and 22 closed-ended questions was prepared to collect information regarding the treatment of EPL among dentists in Spain.

This anonymous questionnaire comprised data in relation to the following (Supplement 1) 

(http://www.medicinaoral.com/medoralfree01/aop/jced_61130_s01.pdf) demographic details, qualification, and work experience, most commonly performed therapy in EPL, sequence of periodontal and endodontic treatment, use of antibiotics, and treatment success rate. Also, opinion about the evidence of this topic and its application into everyday clinical practice was registered. Each subject could only be answered once, and all data were anonymized.

Professionals who declared more than five years of inactivity were excluded.

-Setting

The questionnaire was distributed through corporate mail to the teaching staff of the UPV/EHU Degree in Dentistry, through social networks (Instagram, WhatsApp), through scientific associations such as the Spanish Society of Periodontology (SEPA), and the professional associations of different autonomous communities in Spain; as well as collaborating professional associations from Asturias, the Basque Country, Cantabria, Galicia and Madrid (Spain).

In addition, participants were asked to spread the survey to other dentists to reach a larger number of clinicians. Instructions about how to answer the questionnaire, together with a brief message describing the objectives of the study and its scientific and epidemiological outcomes were also highlighted. The online questionnaire was closed to the public on the 28th of February (2022), and data collection was automatically gathered via the www.googleforms.com server.

-Statistical analysis

The collected data were analyzed by an experienced and blinded statistician (XMM) using IBM SPSS® Statistics 22.0 software (IBM, Chicago, IL, USA). For descriptive categorical variables we used percentages, and to determine the statistical relationship between periodontist and endodontist participants, the Fisher’s exact test or Pearson’s chi-square test was applied. In all cases, only *p-value*s <0.05 were considered statistically significant.

-Bibliographic review

We performed a literature review on the treatment of EPL in three electronic databases (PubMed, Web of Science, and Scopus) using the combination of the following keywords: (“endoperiodontal lesion”) OR (“endo-periodontal lesion”)) AND (“treatment”). We took mean clinical attachment gain (CAG) as the main study variable for the different EPL treatments. Inclusion criteria of the studies were as follows: 1) being written in English or Spanish in the last 20 years, 2) including patients whose follow-up was at least 4 months. On the contrary, exclusion criteria of the studies were: 1) not describing the EPL treatment, 2) not presenting the baseline or final data, and 3) not being possible to calculate the post-treatment CAG.

## Results

-Questionnaire Survey

All the results obtained from the survey are featured in Table 1- Table 1 cont.-2.

**Table 1:**
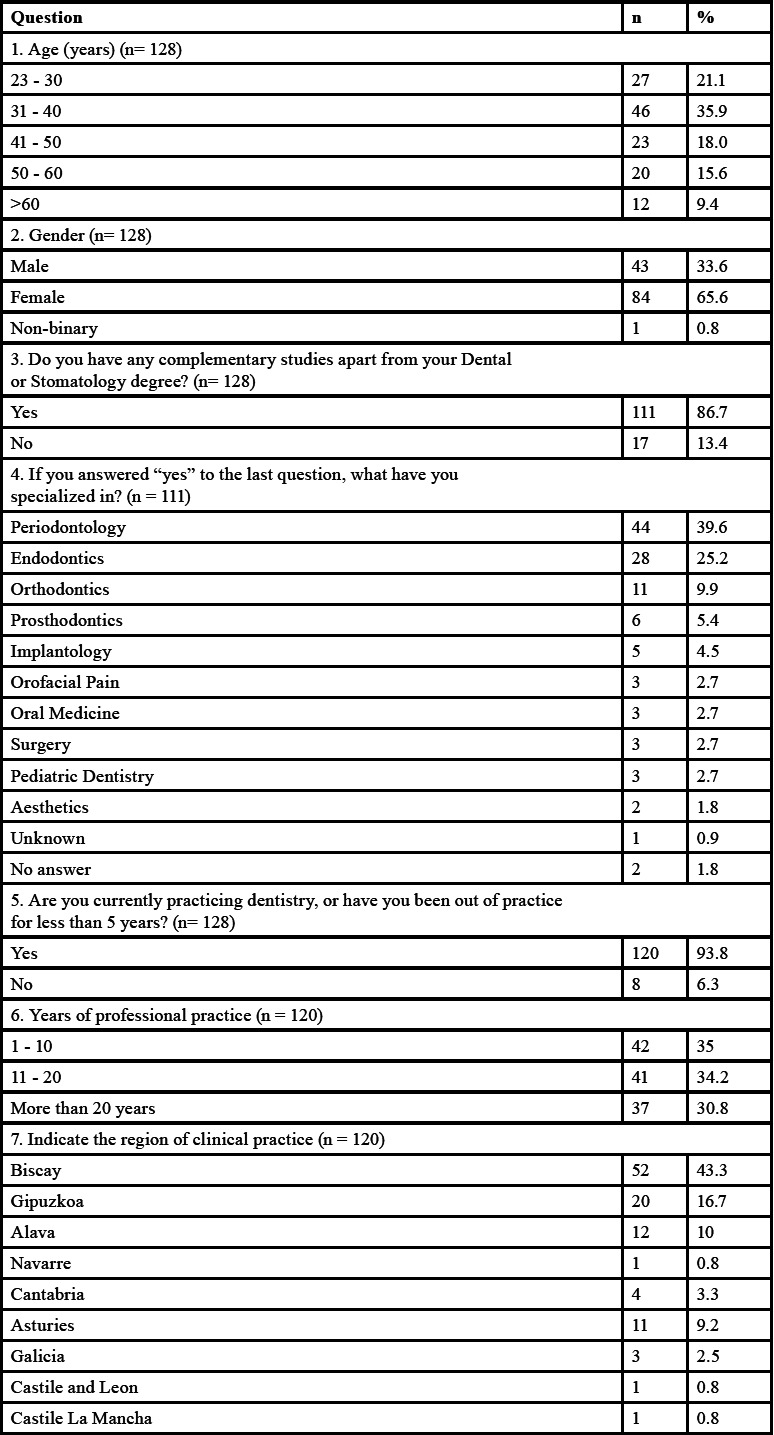
Survey outcome variables: n: Frequency, % percentage, *: open answer question.

**Table 1 cont.**
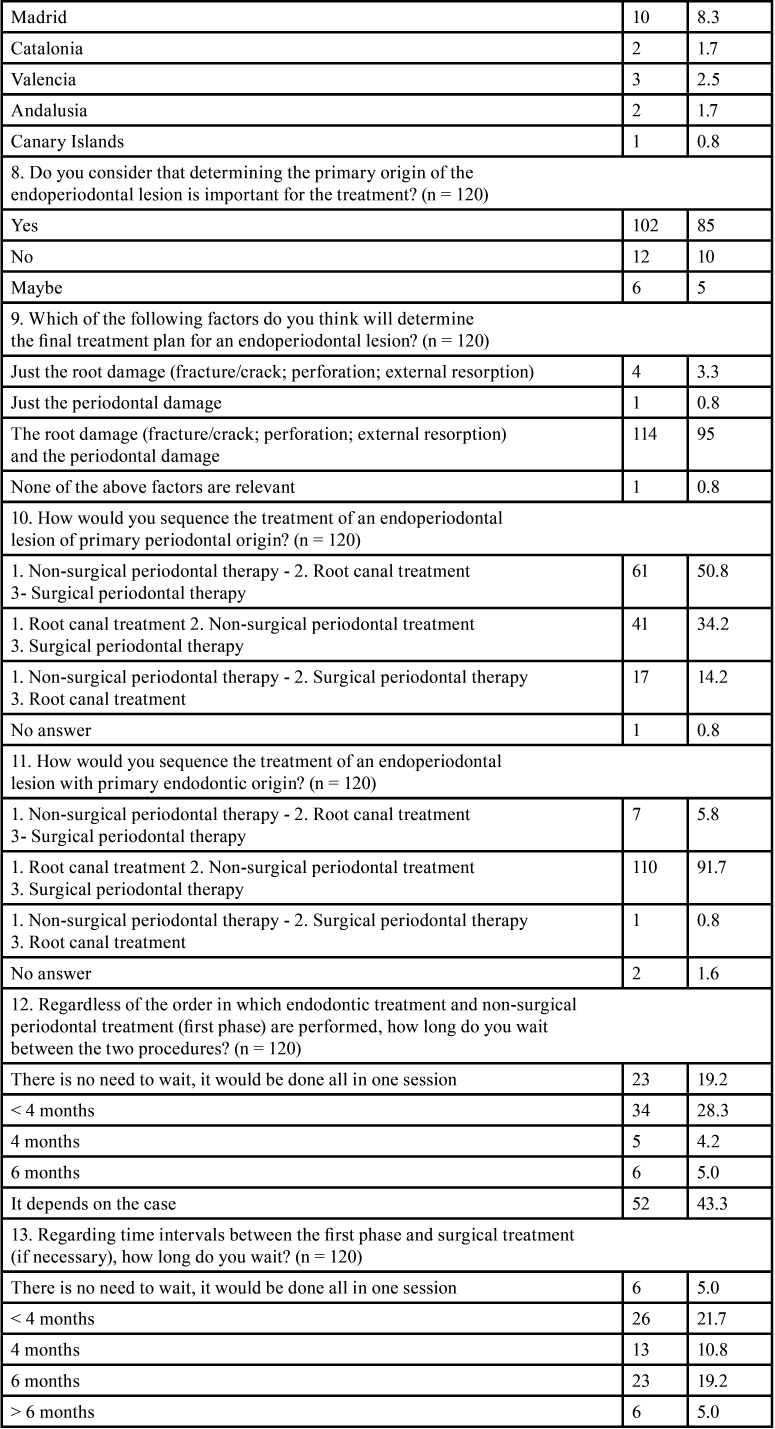
Survey outcome variables: n: Frequency, % percentage, *: open answer question.

**Table 1 cont.-1:**
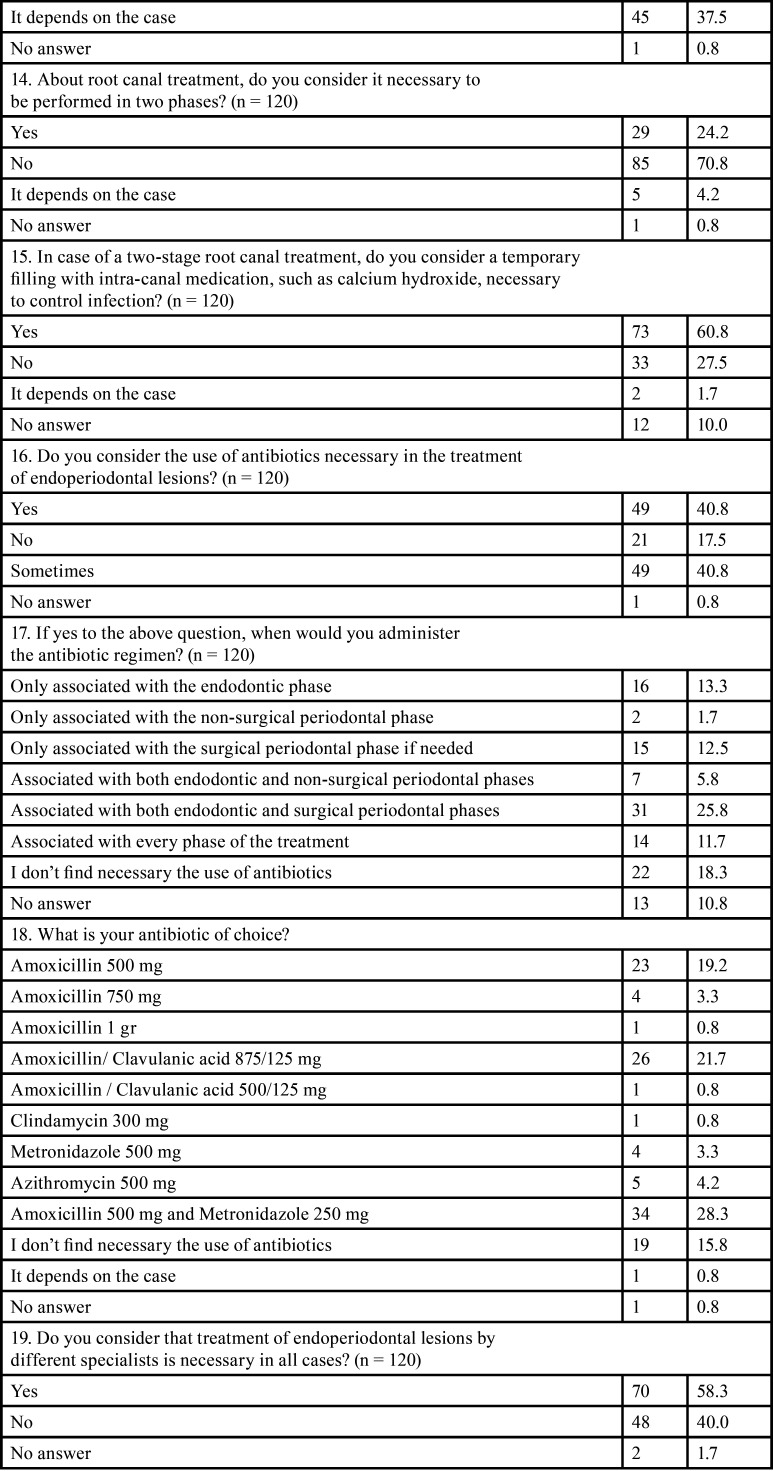
Survey outcome variables: n: Frequency, % percentage, *: open answer question.

**Table 1 cont.-2:**
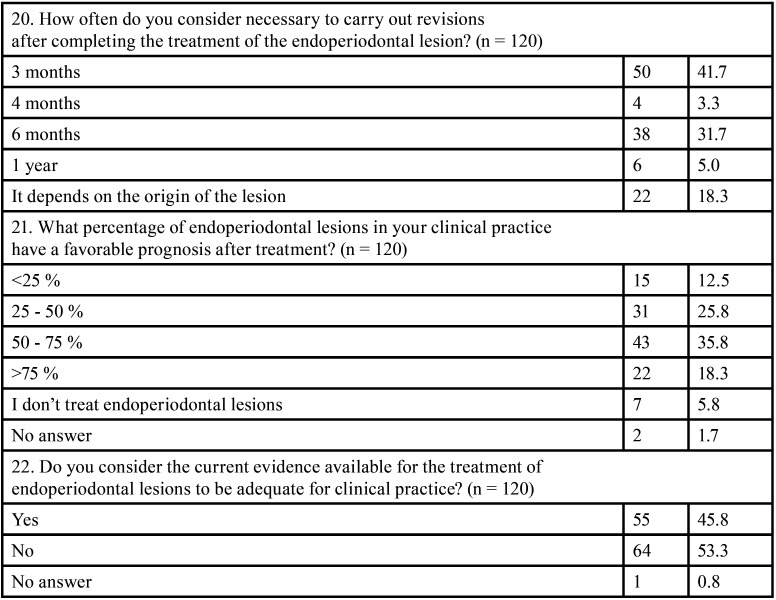
Survey outcome variables: n: Frequency, % percentage, *: open answer question.

Initially, a total of 128 responses were obtained between November 2021 and February 2022, out of which 65.6% were women, with an age range between 31 and 40 years (35.9%). In total, 86.7% of participants had completed complementary studies to the degree/licensure in Dentistry or medical specialization in Stomatology; with periodontics (39.6%), endodontics (25.2%), and orthodontics (9.9%) being the most common specialties among the respondents. One of the conditions for participating in the survey was to be in active practice or at most five years without practicing; thus, 120 subjects finally completed the survey. A total of 43.3% of the surveyed professionals worked in Biscay.

Regarding the factors to be considered prior to therapy, 85% of the respondents stated that it was important to determine the primary origin of the lesion in order to establish the treatment plan. Thus, root damage and the periodontal status of the tooth (95%) were considered the most relevant factors for the respondents.

**Figure 1:**
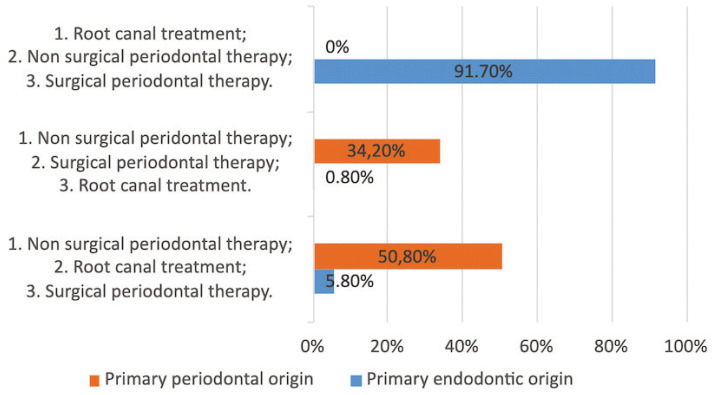
Representative graphic of the answers about the treatment plan for endoperiodontal lesions.

When analyzing the treatment sequence (4), 50.8% of the participants chose non-surgical periodontal therapy (NSPT) as the first therapeutic option for a primary periodontal EPL, followed by root canal treatment, and then surgical periodontal phase (if necessary). On the other hand, when faced with an EPL of primary endodontic origin, 91.7% chose root canal therapy as the initial treatment option, followed by the NSPT and finally the surgical periodontal phase (Fig. 1).

According to specialty, no statistically significant differences were observed in the therapeutic approach between the sequences performed by periodontists (n = 44) and endodontists (n = 27). Neither in the approaches to periodontal (*p* = 0.17) or endodontic (*p* = 0.643) treatment of these lesions. However, in the management of EPL of periodontal origin, 34.1% of periodontists and 25.9% of endodontists started with root canal treatment, followed by NSPT and finally, surgical periodontal treatment (if necessary).

Regarding the time between the different treatments (time interval), the majority of respondents believed this to be case-dependent, both between the initial phase (endodontic/periodontal treatment, regardless of the order) (43.3%) and between the non-surgical and surgical phase (37.5%). Also, 28.3% and 21.7% of respondents indicated that the time interval should be less than 4 months between the initial phases (endodontic and/or periodontal) and between them and surgical periodontal treatment, respectively.

In the endodontic phase of EPL treatment, 70.8% of the participants thought it was not necessary to perform it in two phases. Nevertheless, if performed in two phases, 60.8% of the respondents would use an intra-canal medication to control the infection, while 27.5% did not think it was necessary.

Regarding the use of antibiotics during the treatment of EPL, 40.8% reported that their use was always necessary, and 40.8% that only sometimes; with amoxicillin being the first antibiotic of choice (74.1%), although in different dosages and sometimes associated with other drugs. For the application phase, 25.8% considered that antibiotic therapy should be associated with the endodontic and surgical periodontal phases, and 13.3% only with the endodontic phase.

With all of the above, 58.3% of the respondents referred that the treatment of these lesions should to be interdisciplinary. Nonetheless, there was a statistically significant difference between periodontists (no = 25% / yes = 75%) and endodontists (no = 51.9% / yes = 48.1%) (*p* = 0.039) in this regard.

The responses on the prognosis and follow-up of these lesions after treatment were not homogeneous, and ranged from 3 (41.7%) to 6 months (31.7%). A favorable prognosis was observed in more than 50% of the lesions treated by 54.1% of the participants compared to the 38.3% of the respondents whose treatment success was less than 50%.

Finally, 53.3% of the participants reflected that the available evidence regarding the treatment of EPL was not adequate; versus 45.8%, who stated that it was. Furthermore, comparing the point of view of both periodontists and endodontists, no statistically significant difference was found (*p* = 0.318). However, there were more periodontists (n = 30) than endodontists (n = 15) who considered the available evidence to be scarce.

-Bibliographic review

A total of 69 articles were identified on electronic databases (15 PubMed, 36 Scopus and 18 Web of Science), as well as 17 through manual research. After abstract/full-text review, 9 studies (10-18) were selected.

According to these, 289 EPL were treated with a follow-up between 6 (11,17) and 216 (14) months. Single therapy with endodontic treatment got a mean CAG of 2.33 mm (15,16). Combination protocols proposed for EPL therapy included the endodontic treatment alongside: 1) non-surgical periodontal therapy (NSPT) (CAG= 4.57 mm) (2,11) NSPT and periodontal surgical therapy (CAG= 5.22 mm) (3,17,18) Guided tissue regeneration (GTR) (CAG= 6.75 mm) (10,12,13,23), and 4) NSPT and plastic periodontal surgery (CAG=10 mm) (10,14).

-Proposal of a clinical protocol

**Figure 2:**
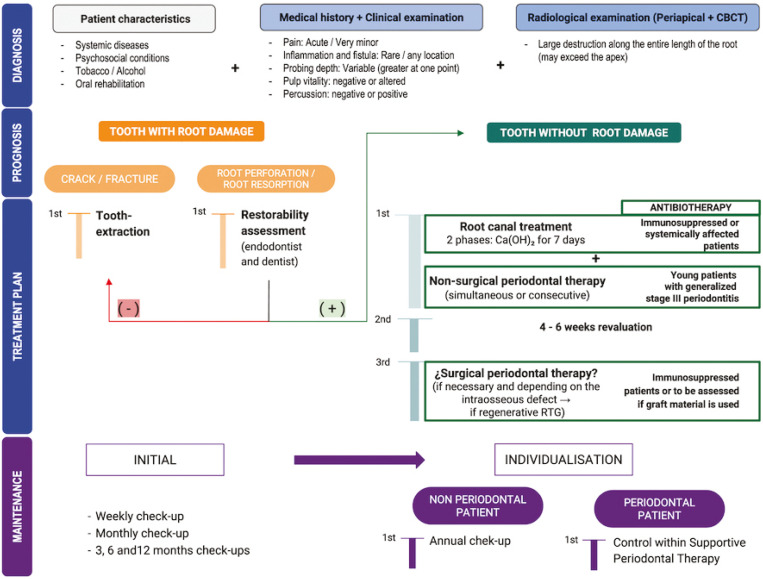
Proposed clinical protocol for the approach of endoperiodontal lesions. Diagram of decision-making, diagnosis and prognosis, treatment and maintenance program according to the clinical characteristics of the tooth and the patient.

Based on the findings obtained in the survey and the current available evidence (10-18), the following clinical protocol is proposed, divided into three phases of action based on the diagnosis, prognosis and treatment plan (Fig. 2).

First, a correct diagnosis of the pathology to be treated will be made, assessing the patient’s signs and symptoms, together with clinical and radiographic parameters (parallel technique).

Secondly, the final diagnosis will be established following the AAP 2018 classification (1), with the aim of determining the prognosis of the tooth, the tooth-root status, its vitality and level of residual attachment, as well as the periodontal status of the patient (1).

In the case of a lesion in a tooth with root damage, it will be necessary to evaluate its origin. When a fissure or root fracture is detected, the first treatment option will be exodontia, due to the hopeless prognosis of the tooth (19). On the contrary, when the root damage is due to a root resorption or perforation, the restorability of the tooth should be assessed by means of a medical interconsultation with the endodontist and the dentist, to determine the viability of the treatment.

After the decision has been made to maintain the tooth, a full-mouth periodontal assessment must be performed to evaluate the periodontal status. In the case of an EPL in a patient without periodontal pathology, the first phase of treatment will be the performance of root canal treatment simultaneously with the NSPT of the affected tooth (tartrectomy and scaling and root planing), or consecutively, reducing the waiting interval between phases to a minimum (between seven and ten days maximum). In the case of an EPL in a periodontal patient, the sequence will be the same, with NSPT being applied to the full mouth (including the hygienic phase).

In relation to root canal treatment, this should be carried out in two phases due to its microbiological complexity, ideally using calcium hydroxide for seven days between instrumentation and obturation of the canals, in order to eliminate the highest percentage of pathogens possible.

Subsequently, a prudent time interval will be defined to ensure the regeneration of the lost tissues associated with the pulp component of the lesion, in order to evaluate the real need to carry out surgical periodontal therapy. The surgical periodontal phase will be performed when presenting a PD >4 mm with bleeding on probing, in addition to a plaque control of less than 25% in the re-evaluation of the affected tooth. Taking the most current evidence as a reference (17), we consider that a period of time between 4-6 weeks would be enough to achieve resolution of the lesion. At this point, the type of surgical periodontal treatment to be performed will be determined by the characteristics of the intraosseous defect present (number of walls and angulation, extension and width of the bone defect). In the case of a regenerable defect, GTR will be the procedure of choice, together with minimally invasive management of the soft tissues in order to reduce soft tissue morbidity and the presence of post-surgical gingival recession.

One of the most important factors in ensuring the long-term treatment success for EPL will be the tailored supportive periodontal treatment (SPT) based on individual clinical characteristics. Ideally, weekly, monthly, three-month, six-month and twelve-month follow-ups will be performed. Subsequently, dental re-evaluation will be specifically included in a STP program in periodontal patients; while in non-periodontal patients it will be necessary to establish a tailored maintenance program with annual check-ups to assess the stability of long-term results.

## Discussion

EPL is an infectious inflammatory pathology that originates as a consequence of a physiological or non-physiological microbial communication, mainly Gram (-) anaerobic bacteria, between the pulp and periodontal tissues (20,21). The prognosis of non-physiological cases is uncertain, generally impossible (22).

The scientific evidence currently available is poor (10-18), coinciding with the perception of more than half of the Spanish professionals that we surveyed, which makes it difficult to make decisions in daily clinical practice. Classically, it has been considered essential to know the primary origin of physiological EPL (23), similar to what was observed in 85% of our participants.

Differential diagnosis and determination of the primary origin of the lesion are complex, due to the common symptomatology of the lesions. However, it has not been considered a key circumstance (24), as both the periodontal and the pulp lesion need to treated. On the other hand, it is key to determine the prognosis of the affected tooth at the time of clinical examination, as well as the stage and severity of the periodontal lesion itself (25). Thus, the prognosis of the tooth will depend mainly, in addition to its root condition, on the initial attachment loss and the number of root canals of the tooth, the presence of periodontitis and the patient’s smoking habit (26).

In relation to the treatment protocols and sequences, most of the participants reported starting with root canal treatment when the primary origin of EPL was endodontic, matching with the current literature (10-18,24). However, when the origin was periodontal, the survey results showed greater heterogeneity, where more than half of the dentists started treatment with the NSPT, without finding statistically significant differences between periodontists and endodontists.

Regarding the waiting period between the different phases of treatment, the outcomes of the survey were heterogeneous, similar to previous studies (10-18,20,27); where different waiting intervals between the endodontic and periodontal phases have been described: 0 (10,14,17,18) and 6 months (27). However, in our study, the highest percentage (43.3%) of participants considered that this interval depends on the characteristics of the EPL. Previous studies indicate that the wait for the surgical phase ranged from one week (10) to four months (14). In our case, most of the professionals (37.5%) considered that this period relies on the characteristics of the lesion, followed by 21.8% who considered that a time interval of less than 4 months would be enough.

A noteworthy aspect of our survey results was the use of adjuvant antibiotics for the treatment of EPL. A total of 40.8% of the clinicians considered its administration necessary and another 40.8% that depended on the type of lesion. These findings contrast with current guidelines, which justify its use together with periodontal treatment only in patients with stage III grade C periodontitis (1), and EPL are not among the situations included in the antibiotic use guidelines of the Spanish Association of Endodontics (AEDE) approved in 2020 (28). With all this, we believe that the tendency among dental professionals in Spain is to administer more antibiotics than actually necessary. This situation should begin to be controlled, adjusting to the recommendations referred by different scientific associations (EFP and AAP), to try to reduce bacterial resistance, which is currently on the increase due to the abusive use of antibiotics (1,27-30).

It is also striking that 38.3% of the respondents reported a success rate lower than 50% in the EPL treatment. This could be related to the lack of scientific evidence reflected by the professionals, which would prevent the correct management of EPL in daily clinical practice.

Finally, we must recognize some limitations during the performance of the study. Firstly, the refusal of some associations to disseminate the survey among their professional members due to internal regulations could have influenced the limited number of responses received (n = 128). And secondly, the large number of surveys that dental health professionals receive in their daily activities, which could have triggered their acceptation of ours.

Among the strengths of this study, we should highlight that is the first study carried out with dental professionals in Spain regarding the treatment of EPL that proposes a clinical protocol based on the diagnostic phases and treatment planning sequence.

In conclusion, the scientific evidence regarding EPL is currently scarce and heterogeneous in terms of treatment guidelines and expected clinical results, similar to the results of the survey.

Most of the professionals chose endodontic treatment when the primary origin of the lesion was pulpal; while only half started with non-surgical periodontal treatment when the primary origin was periodontal.

Based on our results, we propose a clinical protocol based on the diagnosis of the lesion and a sequenced treatment planning. However, further studies on this type of lesion with larger population samples and long-term follow-up are needed to validate our protocol.
